# Genotype Imputation in Winter Wheat Using First-Generation Haplotype Map SNPs Improves Genome-Wide Association Mapping and Genomic Prediction of Traits

**DOI:** 10.1534/g3.118.200664

**Published:** 2018-11-16

**Authors:** Moses Nyine, Shichen Wang, Kian Kiani, Katherine Jordan, Shuyu Liu, Patrick Byrne, Scott Haley, Stephen Baenziger, Shiaoman Chao, Robert Bowden, Eduard Akhunov

**Affiliations:** * Department of Plant Pathology, Kansas State University, Manhattan, KS 66506; † Texas A&M AgriLife Research, Amarillo, TX 79106; ‡ Department of Soil & Crop Sciences, Colorado State University, Fort Collins, CO 80523; § Department of Agronomy and Horticulture, University of Nebraska, Lincoln, NE 68583-0915; ** USDA-ARS Cereal Crops Research Unit, Fargo, ND 58102-2765; †† USDA-ARS Hard Winter Wheat Genetics Research Unit, Kansas State University, Manhattan, KS 66506

**Keywords:** Wheat HapMap, SNP, Imputation, GWAS, Genomic Prediction, GenPred, Shared Data Resources

## Abstract

Genome-wide single nucleotide polymorphism (SNP) variation allows for the capture of haplotype structure in populations and prediction of unobserved genotypes based on inferred regions of identity-by-descent (IBD). Here we have used a first-generation wheat haplotype map created by targeted re-sequencing of low-copy genomic regions in the reference panel of 62 lines to impute marker genotypes in a diverse panel of winter wheat cultivars from the U.S. Great Plains. The IBD segments between the reference population and winter wheat cultivars were identified based on SNP genotyped using the 90K iSelect wheat array and genotyping by sequencing (GBS). A genome-wide association study and genomic prediction of resistance to stripe rust in winter wheat cultivars showed that an increase in marker density achieved by imputation improved both the power and precision of trait mapping and prediction. The majority of the most significant marker-trait associations belonged to imputed genotypes. With the vast amount of SNP variation data accumulated for wheat in recent years, the presented imputation framework will greatly improve prediction accuracy in breeding populations and increase resolution of trait mapping hence, facilitate cross-referencing of genotype datasets available across different wheat populations.

Chromosomal segments sharing common ancestry in a population, referred to as segments of identity-by-descent (IBD), may be detected using high-density marker data and this information may be used for predicting unobserved genotypes for markers located in the same IBD segment (Browning and Browning 2012). This genotype imputation procedure allows the interpolation of diversity data across diverged populations genotyped using common marker platforms. The imputed genotypes increase the number of SNP available for marker-trait association analyses and genomic prediction, thus increasing the resolution and power of association mapping studies and model-based predictions (Howie *et al.* 2009, Crossa *et al.* 2014). In humans and maize (Abecasis *et al.* 2010; Swarts *et al.* 2014), the accuracy of imputation correlated with the frequency of imputed alleles in a population. It was shown that by increasing the genotyping probability (GP) cutoff value, the accuracy of imputation can be increased at the cost of increasing the proportion of missing genotypes in the final dataset. Genotype imputation performed using the 1,000 human genomes dataset made it possible to test the majority of common variants in a population for marker-phenotype associations (Abecasis *et al.* 2010). It was demonstrated that more complete ascertainment of SNP variation achieved by imputation helped to identify previously unidentified candidate SNP (Huang *et al.* 2012). Similarly, imputation performed in *Arabidopsis* using the whole-genome sequence data generated from a reference panel of 80 strains (accessions) increased the power and resolution of trait mapping in genome-wide association studies (GWAS) (Cao *et al.* 2011). In maize, GBS markers were effectively used to detect the IBD regions in a diverse panel of lines and to predict missing genotypes present at high frequency in this type of genotyping data (Swarts *et al.* 2014).

Until recently, the complexity of the wheat genome was one of the main obstacles for obtaining high-density genetic variation data. As a result, many wheat scientists have relied on low-density PCR-based markers for disease diagnosis and marker assisted selection (Helguera *et al.* 2003; Ullah *et al.* 2016; Xu *et al.* 2018). Several PCR-based markers such as S19M93, S23M41, *Xpsp*3000, Iag95 and VENTRIUP-LN2 are commonly used in screening of wheat lines for stripe rust resistance (Ullah *et al.* 2016). However, the development of genotyping arrays (Cavanagh *et al.* 2013; Wang *et al.* 2014b) and next-generation sequencing-based methods of genotyping (Saintenac *et al.* 2011; Poland *et al.* 2012; Saintenac, Zhang, *et al.* 2013; Jordan *et al.* 2015) has enabled the generation of SNP markers covering the entire wheat genome. Large populations of diverse lines and recombinant inbred wheat lines have been genotyped using the 9K and 90K SNP arrays, and used for mapping a number of traits related to disease resistance (Sela *et al.* 2014; Maccaferri *et al.* 2015), yield (Kalous *et al.* 2015), height (Zanke *et al.* 2014a), heading date (Zanke *et al.* 2014b), and domestication (Faris *et al.* 2014). The GBS method has become the technology of choice for cost-effective genotyping and trait mapping (Saintenac, Jiang, *et al.* 2013; Crespo-Herrera *et al.* 2014; Li *et al.* 2015). While GBS is characterized with high missing genotype data, imputations approaches have enabled the use of these markers for genomic prediction and genome-wide association studies in various species including wheat (Crossa *et al.* 2016). Finally, a high-density haplotype map of wheat was generated using the whole exome capture and GBS, which provided a detailed description of the majority of common variants in the genic regions of the wheat genome at the sequence level (Jordan *et al.* 2015).

With the development of a first genomic reference sequence (International Wheat Genome Sequencing Consortium 2014) it has become possible to use the wheat SNP variation maps for IBD prediction and imputing missing genotypes in diverse collections of lines. Here, a first-generation wheat SNP haplotype map was used to develop a resource for imputing marker genotypes in populations previously genotyped using either the 90K SNP iSelect wheat array (Wang *et al.* 2014b) or GBS (Poland *et al.* 2012). The utility of GBS and 90K SNP datasets for imputing ungenotyped markers was assessed. We used SNP genotyped by exome capture in the reference panel of 62 wheat lines (Jordan *et al.* 2015) to impute ungenotyped markers in a diverse collection of winter wheat cultivars from the U.S. Great Plains. It was demonstrated that SNP from the reference panel can be accurately imputed into the winter wheat population, significantly increasing marker density to improve the accuracy of genomic prediction and the number of detected marker-trait associations in GWAS.

## Materials and Methods

### Diversity panel and genotyping

The association mapping panel of hard red winter wheat cultivars (henceforth, WWAM panel) included 307 accessions from major breeding programs across the U.S. Great Plains (Table S1). The WWAM panel was genotyped using the custom 90K SNP iSelect wheat array and GBS according to Saintenac *et al.* (2013). The GBS data were generated for the WWAM panel by sequencing the barcoded 96-plex libraries on a single lane of HiSeq2500 instrument. Raw GBS reads of 100 bp in length were trimmed to remove low quality bases (quality < 15) from both ends retaining only reads of > 30 bp. A filter was applied to select reads with at least 80% of bases having quality >15. Reads passing the quality control steps were separated based on the barcode sequences corresponding to individual accessions. GBS data were aligned to the assemblies of the wheat flow-sorted chromosomes (International Wheat Genome Sequencing Consortium 2014) using a previously described mapping strategy (Jordan *et al.* 2015). In brief, the strategy uses the Bowtie program (Langmead and Salzberg 2012) with three sets of alignment parameters with decreasing mapping stringency. Reads not aligned at higher stringency levels were re-used at lower stringency levels.

The read alignment files generated for each accession were sorted and indexed using SAMtools (Li *et al.* 2009) prior to variant calling. The program Picard v. 1.62 (https://sourceforge.net/projects/picard/files/picard-tools/1.100/) was used to remove duplicated reads. Variant calls were generated using the UnifiedGenotyper module of genome analysis toolkit (GATK) v2.2 with default parameters (McKenna *et al.* 2010). Variant calls were filtered to remove sites with > 50% missing genotype data and minor allele frequency (MAF) of < 0.03.

A cleaved amplified polymorphic sequence (CAPS) marker (Ventriup) linked with the *Yr17* stripe rust resistance gene was used to confirm the presence of this gene in the WWAM panel (Helguera *et al.* 2003). The *Yr17* gene was shown to be present in cultivar Jagger broadly used in the breeding programs across the U.S. Great Plains (Fang *et al.* 2011). PCR products were analyzed on an ABI PRISM 3730 DNA Analyzer (Applied Biosystems, Foster City, CA). Data collected were processed using GeneMarker v1.6 (SoftGenetics LLC, State College, PA) followed by visual validation of genotype calling accuracy.

### Imputation of missing SNP markers using exome capture SNP from the reference panel

**Figure 1:**
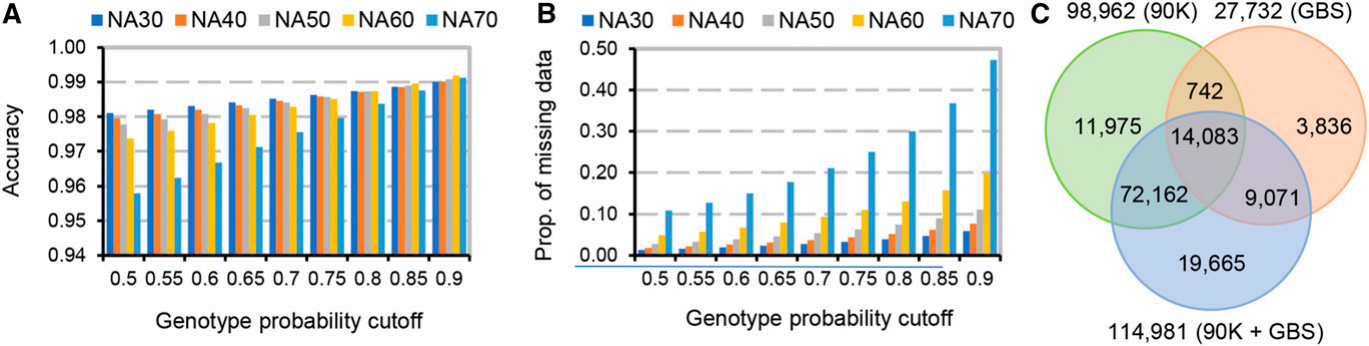
Genotype imputation using the exome capture SNP. Impact of genotype probability cutoff value on the accuracy (A) and the proportion of missing genotypes (B) in the imputed datasets. NA30, NA40, NA50, NA60 and NA70 correspond to simulated SNP containing 30%, 40%, 50%, 60% and 70% of genotypes missing, respectively. C. The number of imputed SNP in the WWAM panel genotyped using different approaches.

We tested two different genotype imputation scenarios in the WWAM panel. In the first one, we predicted sporadically missing markers at genotyped loci in the WWAM panel using both the 90K SNP iSelect array and GBS SNP (Figure 1A, 1B). In the second scenario, the 90K SNP array and/or GBS markers shared between the target WWAM panel and the reference panel of 62 wheat lines (Jordan *et al.* 2015) were used to impute ungenotyped SNP sites in the WWAM panel.

To test the accuracy of imputation at genotyped SNP sites with missing data in the WWAM panel, we generated a subset of genotype dataset containing 15,686 SNP selected from the 90K SNP iSelect array, including 5,618, 8,210 and 1,858 SNP mapped to the A, B and D genome, respectively. These data were used to generate random samples with different proportions of missing genotypes. A total of 10 datasets for each level of missing data were generated. The imputed genotypes were then compared to the original genotypes to calculate the accuracy of imputation.

For imputing missing SNP sites in the WWAM panel that were genotyped in the reference panel of 62 wheat lines, we used three sets of SNP markers: 1) markers genotyped using the 90K SNP iSelect array, 2) GBS markers, and 3) combined 90K SNP iSelect and GBS markers. A total of 9,786, 4,876, and 14,662 markers overlapping between the target WWAM population and the reference panel were selected from each of these three datasets, respectively. These sets of markers for imputation using the 62 accessions reference panel are available for download from the wheat HapMap project page (http://wheatgenomics.plantpath.ksu.edu/hapmap/).

The program Beagle v4.0 (Browning and Browning 2013) was used to impute missing and ungenotyped SNP markers in the target population of winter wheat cultivars using exome capture SNP from the reference panel of 62 wheat lines. The imputation parameters included “nthreads=10 burnin-its=10 phase-its=10 window=5000 overlap=500”, as previously described by Jordan *et al.* (2015). The imputed files were filtered using customizable cut-off values of genotyping probability (GP; default value = 0.8), proportion of missing genotypes (default value = 0.5) and minor allele frequency (MAF; default value = 0.03). SNP genotyped using the 90K iSelect wheat array or GBS or both were used for detecting the IBD regions between the reference and target population.

To facilitate imputation, we have developed a set of scripts for formatting 90K and GBS genotype data generated for any target population. The imputation workflow is shown on Fig. S1 and scripts can be downloaded from the following website: http://wheatgenomics.plantpath.ksu.edu/hapmap/. The input files can be provided in either the VCF (Variant Call Format) or hapmap format. Perl scripts were developed to transform data from the hapmap to the VCF format used by Beagle v4.0. These scripts and some example data to test-run the scripts can also be downloaded from GitHub website: https://github.com/wheatgenomics/haplotypeV1_imputation. Currently, the input SNP should have the coordinates based on the wheat flow-sorted chromosome survey sequencing (CSS) contigs (International Wheat Genome Sequencing Consortium 2014). However, to facilitate the imputation of SNP generated based on the new version of wheat reference sequence (International Wheat Genome Sequencing Consortium 2018), we updated the SNP positions for the reference panel. This was achieved using Perl scripts and SAMtools to extract 50 bp of sequences flanking the SNP on both sides from the contigs of CSS. For SNP that were located at less than 50 bp from the start of the contig, the start position of the flanking sequence was adjusted to one. The extracted sequences were aligned to the CS reference using blastn. The blastn output file was filtered on percent identity, e-value, mismatches and unique alignment using a Perl script. Only sequences that had 100% identity with the reference, without any mismatch, evalue ≥ 3.45e-45 and had only one hit in the entire reference sequence were retained. SNP positions were then calculated by subtracting 50 from end position of the sequence alignment. The new ordering was then linked to the vcf files containing the reference panel SNP using a Perl script. These updated SNP can be accessed from the website: http://wheatgenomics.plantpath.ksu.edu/hapmap/.

The DNA strand designation and orientation of allele calls provided for the target population were compared with that of the reference population. Allele designation and reference allele frequency were used to determine whether the target and reference chromosome strands were identical or not. In case there was no match between the reference and target population, the designation and orientation of alleles were corrected. A Perl script was developed to check the consistency between the target and reference VCF files (http://wheatgenomics.plantpath.ksu.edu/hapmap/). Users may also use the program “conform-gt” for that purpose (https://faculty.washington.edu/browning/conform-gt.html).

### Phenotyping

The WWAM panel was evaluated in the greenhouse and in field conditions for resistance to *Puccinia striiformis* f.sp. *striiformis* (Pst), a fungal pathogen causing wheat stripe rust. Three field experiments to evaluate adult plant stripe rust resistance were conducted in 2010 and 2011 in Rossville, KS, and in 2012 in Castroville, Texas (Table S2) using a completely randomized design (CRD) with three replicates. Approximately 30 seeds were sown per 2 m rows spaced 25 cm apart. To provide a uniform level of infection, spreader rows of susceptible wheat line (KS89180B-2-1) were planted every 5^th^ row and infected with the PST-100 race of Pst. Infection type (IT) was scored using the McNeal 0-9 scale (McNeal *et al.* 1971), and severity of infection (SV) was assessed using the modified Cobb 0-100 scale (Peterson *et al.* 1948), when the disease severity on a susceptible line reached 60–70%.

Adult plant and seedling stage resistance to stripe rust were also evaluated in greenhouse conditions using the previously described procedures (Hulbert *et al.* 2007). Ten day-old wheat seedlings or anthesis-stage adult plants were inoculated with urediniospores of race PST suspended in Soltrol 170 mineral oil (Chevron-Phillips Chemical Company, The Woodlands, TX) and then placed in a dew chamber for 16 h in the dark at 12-15°. Seedlings were then transferred to a growth chamber with 16 h photoperiod and maintained at 15 ± 1° day and 12 ± 1° night temperatures. Seedlings were scored for IT at 18 days after inoculation using the McNeal scale. Adult plants were transferred to a greenhouse with 16 h photoperiod and maintained at 18 ± 3° day and 13 ± 3° night temperatures. Adult plants were scored for IT and SV at 21 days after inoculation as described above.

Analysis of variance components and Pearson correlation coefficients were calculated using the base functions of R. Broad sense heritability (*H*^2^) was estimated as

$$H^{2}=\frac{V_{G}}{V_{G}+V_{E}}$$

Where *V_G_* is genetic variance and *V_E_* is environmental variance components extracted from ANOVA results.

A linear mixed effect model with restricted maximum likelihood implemented by R package lme4 was used to fit the phenotype data: $y_{ij}=\mu +G_{i}+E_{j}+GE_{ij}+\varepsilon_{ij}$, where *y_ij_* is the phenotype of the *i^th^* wheat cultivar in *j^th^* location, *μ* is the intercept, *G_i_* is the *i^th^* cultivar, *E_j_*is the *j^th^* location, *GE_ij_* is the cultivar by location interaction and *ε_ij_* is random residual. Cultivars were considered as random variables while locations were fixed. Best linear unbiased predictions (BLUPs) for cultivars were extracted from the model and used in genomic prediction.

### Genome-wide association analysis

In our study, we used the Empirical Normal Quantile transformation approach implemented as the function “tRank” in the R package “multic” (Peng *et al.* 2007) to assess the impact of different normalization approaches on the results of genome-wide association tests performed using the R package GAPIT (Lipka *et al.* 2012). Comparison of genome-wide association test results performed with the BLUPs and normalized/transformed phenotypic values gave similar results in terms of the significance and number of detected marker-trait associations.

Genome wide association mapping was performed using analysis routines implemented in GAPIT (Lipka *et al.* 2012). The method SUPER (Settlement Under Progressively Exclusive Relationship) was applied for marker-trait association analyses (Wang *et al.* 2014a). The method was shown to retain the computational efficiency of the FaST-LMM model (Lippert *et al.* 2011) and significantly increased power (Wang *et al.* 2014a). The LD threshold in the SUPER method was set to 0.1 (Wang *et al.* 2014a). The first three principal components inferred using GAPIT were used to control for population structure. Marker-trait association was performed for each location. SNP markers that showed significant association in at least two environments were reported.

### Genomic prediction

The predictive ability (PA) of BayesB and reproducing kernel Hilbert space (RKHS) models was used to compare between using SNP genotyped by the 90K iSelect array and GBS (SNP set 1) and SNP set 1 plus imputed SNP (SNP set 2) to predict stripe rust resistance in WWAM panel. The imputed SNP included in set 2 were those that had trait-marker association p-values ≤ 0.05 for infection type to stripe rust. Genotypes were converted to numeric data (0,1 and 2) using a custom R script called AlleleDosage (Nyine *et al.* 2018), where 0 is homozygous major allele, 2 is homozygous minor allele and 1 is the heterozygous state of the alleles at a SNP locus. The script removes monomorphic SNP and loci where the minor and major allele cannot be designated.

The population was randomized and divided into five groups (k = 5) during cross validation. Three independent runs of BayesB and RKHS models were performed resulting in 15 cross validations per trait. The average PA plus the standard error of the mean were calculated for each trait. The effect of increasing SNP markers by imputation on prediction was reported as percent gain. This was calculated as the difference between PA of SNP set 2 and SNP set 1 divided by PA of SNP set 1 and the result multiplied by 100. All models were implemented in the R package BGLR (Pérez and de los Campos 2014) with 10000 iterations, 5000 burnin and 10 thin as Markov chain Monte Carlo parameters.

### Data availability

Sets of markers for imputation using the 62 accessions reference panel are available from the wheat HapMap project page http://wheatgenomics.plantpath.ksu.edu/hapmap/. Sequence data are deposited to NCBI SRA: PRJNA312508.

The Perl scripts used in handling the data and some example data to test-run the scripts can be accessed from GitHub website: https://github.com/wheatgenomics/haplotypeV1_imputation or wheat HapMap project page http://wheatgenomics.plantpath.ksu.edu/hapmap/.

The updated version of SNP for the 62 accessions reference panel based on the new Chinese Spring reference can be access from the website: http://wheatgenomics.plantpath.ksu.edu/hapmap/.

Figure S1 is the workflow for SNP imputation in wheat. File S1.txt contains SNP HapMap used in genome-wide association and genomic prediction. Table S1 contains a summary of GBS data generated for the winter wheat diversity panel. Table S2 shows the phenotypes collected for stripe rust resistance. Table S3 contains the pair-wise Pearson’s correlation coefficient for stripe rust resistance phenotypic values. Table S4 contains the significant marker-trait associations obtained in the GWAS of resistance to wheat stripe rust. Table S5 shows the overlap of identified GWAS signals with SNP previously shown to be associated with resistance to wheat stripe rust. Table S6 shows the association of the Ventriup marker with stripe rust resistance. Table S7 contains a list of SNP significantly associated with stripe rust resistance and showing high LD (r2 > 0.5) with the Ventriup marker. Supplemental material available at Figshare: https://doi.org/10.25387/g3.7294766.

## Results

### Population genotyping

High-density genotype data for the WWAM panel were obtained using the 90K SNP iSelect array (Wang *et al.* 2014b) and the GBS approach (Saintenac, Jiang, *et al.* 2013). A total of 23,562 polymorphic SNP was identified using the 90K array (File S1). More than 736 million quality-filtered reads were generated for the WWAM panel accessions (Table S1). After quality trimming and filtering, 87% of reads (> 640 million reads) could be assigned to individual accessions using barcode sequences (Table S1). On average, about 2.4 million reads with an average length of 82 bp were generated for each accession. Using the previously described alignment strategy (Jordan *et al.* 2015), 640,582,951 reads (87%) were aligned to the CSS reference with 372,351,242 reads (51%) aligning uniquely. Multi-sample variant calling in the GBS dataset using the GATK pipeline (McKenna *et al.* 2010) generated about 9.4 million raw variants. After filtering for sites with a maximum of 50% missing data, we obtained 27,732 SNP.

### Genotype imputation using the wheat exome capture variation data

Results of sporadic imputation of missing genotype data for the WWAM panel using both the 90K and GBS SNP are summarized in Figure 1A and 1B. While the accuracy of imputation was strongly influenced by the proportion of missing genotypes in the datasets, a high accuracy > 95% was achieved even at the sites with up to 70% missing data. The value of GP ≥ 0.8 applied in our study as the lower cutoff value for imputed genotypes in the GBS dataset resulted in > 98% accuracy and < 30% of missing data (Figure 1A).

The total number of imputed ungenotyped SNP for the WWAM population (after filtering with a genotype probability threshold of 0.8, MAF > 0.03 and proportion of missing data per site less than 50%) was 98,962 for the 90K SNP iSelect set, 27,732 for the GBS set and 114,981 for the combined set (Figure 1C). Only 14,083 imputed markers overlapped among all three datasets suggesting that a relatively small fraction of IBD segments could be predicted consistently using markers from either the 90K SNP iSelect array or the GBS datasets. By combining both GBS and 90K SNP it was possible to impute 114,981 genotypes out of which 75% overlapped with those predicted using the 90K SNP iSelect array and 20.1% overlapped with those predicted using GBS. To increase the utility of reference diversity panel for genotype imputation we have developed scripts (http://wheatgenomics.plantpath.ksu.edu/hapmap/) that can help to impute SNP from the reference panel using algorithms implemented in Beagle v4.0 (see Materials and Methods for detailed description).

### Genome-wide association mapping using imputed SNP markers

**Figure 2:**
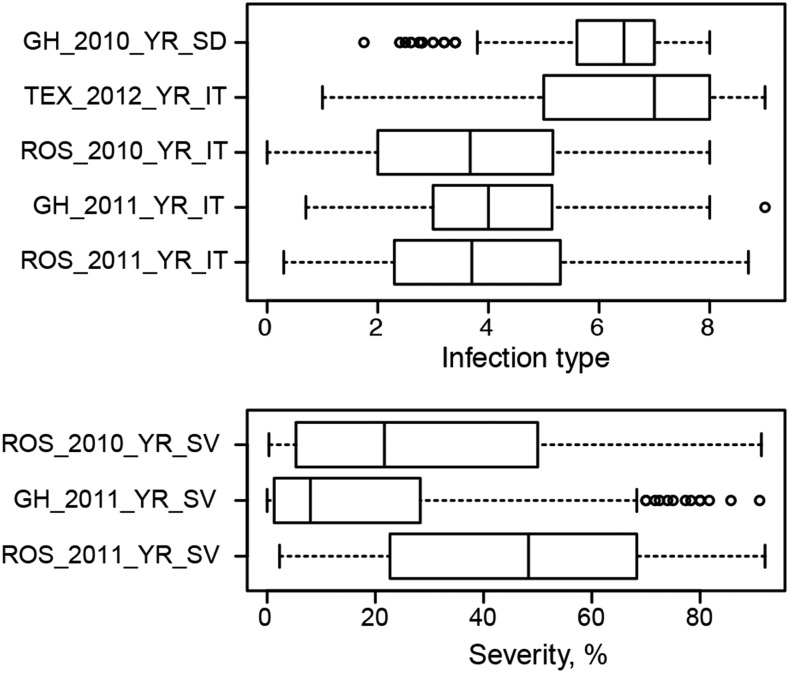
Boxplots of phenotypic data collected for stripe rust resistance for the WWAM panel.

Overall, phenotypic data collected for resistance to stripe rust showed good correlation across different locations and years with the mean Pearson’s correlation coefficient of 0.47 (Figure 2) (Table S3). Broad sense heritability estimates for infection type and severity were 0.76 and 0.77, respectively, consistent with the previous estimates obtained for a diverse panel of wheat landraces (Maccaferri *et al.* 2015).

Resistance to stripe rust measured at the seedling stage showed correlation with the adult plant resistance measured as infection type and severity with correlation coefficients ranging from 0.28 to 0.52 (p-value < 0.05) with the average of 0.39. One exception was the low correlation (*r*^2^ = 0.07) observed between seedling resistance (GH_2010_Yr_SD) and adult plant resistance phenotypes collected during the second round of scoring in year 2011 season at Rossville, KS (ROS_2011_YR_IT).

**Figure 3:**
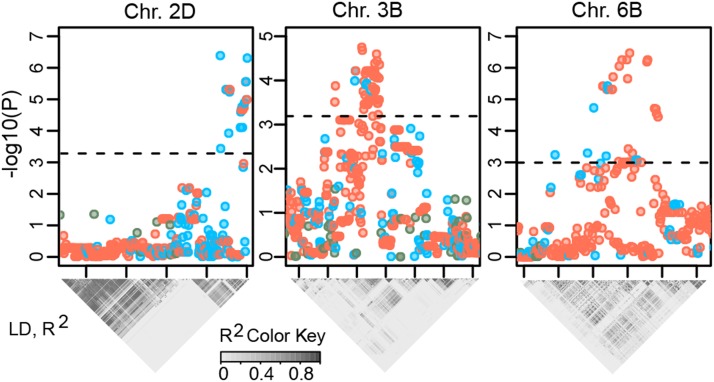
Significance of marker-trait associations using genotyped (blue) and imputed (red) SNP. Pair-wise LD (*r*^2^) between SNP is shown below each chromosomal region.

A total of 146,198 markers (File S1) including both directly genotyped and imputed SNP were tested for association with adult and seedling stage resistance to stripe rust. Neighboring SNP showing significant marker-trait associations at FDR < 0.05 were grouped based on LD (*r*^2^ > 0.3) to estimate the total number of unique loci controlling trait variation in the population. Only those marker-trait associations that were consistent in two different experiments were included into the subsequent analyses. For each region the single most statistically significant SNP was chosen. Comparison of GWAS signals obtained using genotyped and imputed SNP showed that an increase in marker density achieved through imputation can significantly improve the resolution and power of the association tests (Figure 3). The fraction of imputed markers showing significant GWAS signals increased with the decrease of the FDR cutoff value (Pearson’s product moment correlation -0.71, p-value = 0.006) suggesting that the substantial number of imputed SNP are more strongly associated with a trait than directly genotyped SNP.

A total of 17 genomic regions distributed across 13 wheat chromosomes showed significant association with infection type in at least two environments at FDR ≤ 0.05 (Table S4). Five genomic regions on five different wheat chromosomes showed significant association with severity of infection in at least two environments. A total of 12 genomic loci located on 8 chromosomes were associated with disease resistance at the seedling stage. Four genomic regions located on chromosomes 1BL, 2DL, 3B, and 7BL showed marker-trait associations with both severity and infection type suggesting that the same genetic loci may control both traits. Only three genomic regions located on chromosomes 3B, 5AL and 7BL associated with resistance at the seedling stage were associated with severity and infection type at the adult stage suggesting that the majority of identified disease resistance loci confer resistance at the adult stage.

Genomic regions detected for resistance to stripe rust were compared with marker-trait associations mapped in other GWAS. Significant genomic intervals were defined based on the LD blocks around the most strongly associated alleles (Figure 3 and Table S4) and were tested for overlap with previously mapped stripe rust resistance loci. We used the 90K consensus genetic map (Wang *et al.* 2014b) to define intervals harboring GWAS signals considering that LD in the HRWW population decays to *r*^2^ = 0.3, approximately to half of its initial value, at about 2 cM (Chao *et al.* 2010). Out of the 34 genomic intervals, ten overlapped with regions known to harbor SNP contributing to adult and seedling stage resistance to wheat pathogens (Table S5). Among the most significantly associated SNP in each genomic interval, 24 (71%) were imputed SNP. In addition, the Ventriup marker linked with the *Yr17* stripe rust resistance gene was used in marker-trait association analysis. The *Yr17* gene is located on chromosome 2AS and was present in cultivar Jagger, one of the historic cultivars from Kansas broadly used in the breeding programs across the U.S. Great Plains. As expected, the Ventriup marker was significantly associated (FDR < 0.1) with infection type and severity at the adult stage and resistance at the seedling stage (Table S6). The Ventriup marker showed high LD (*r*^2^ > 0.5) with three SNP significantly associated with stripe rust resistance (Table S7). The genotypes of the two of these markers were imputed.

The number of alleles showing a significant positive effect on resistance to stripe rust in at least two experiments with FDR ≤ 0.05 was estimated for each line in the population. The proportion of positive alleles among SNP associated with infection type was correlated with resistance to stripe rust (Pearson’s product-moment correlation = -0.42, p-value ≤ 3.3 × 10^−10^) and these explained about 17.2% of the total phenotypic variance. Similarly, severity level showed strong negative correlation (Pearson’s product-moment correlation = -0.50, p-value ≤ 3.3 × 10^−14^) with the number of positive alleles and explained 17.8% of phenotypic variance. These results suggest that detected loci act additively to confer resistance to stripe rust.

### Genomic prediction of stripe rust resistance traits

The total number of SNP in File S1 converted to numeric data using the AlleleDosage function was 145,605 out of which 114,808 were imputed SNP. The slight reduction in the number of SNP was due to removal of loci where the minor and major alleles could not be determined. Including all imputed SNP in set 2 did not show improvement in prediction accuracy of stripe rust resistance as compared to including only imputed SNP that had a p-value ≤ 0.05 as informed by GWAS. We hypothesized that majority of the imputed SNP were not linked to QTL controlling stripe rust resistance and their minor allele frequency varied greatly causing background noise given the diverse nature of the WWAM panel. The selected imputed SNP were distributed across all chromosomes of the CSS reference. The number of SNP in set 1 and set 2 was 30,797 and 38,123, respectively. The phenotype data used for genomic prediction included mean and BLUP values of cultivars for infection type and severity. For seedling resistance, original observations were used because it was measured once in the green house. Predictions based on BLUPs were slightly better than those based on the mean values of infection type and severity. The gain in prediction accuracy of both BayesB and RKHS models using SNP set 2 was > 50% for infection type and severity relative to using SNP set 1 (Table 1). This significant gain in PA was attributed to the imputed SNP that increased the number of markers in the data set that were associated with the traits. Addition of imputed SNP to SNP set 1 did not improve the prediction of seedling resistance with both models suggesting that the SNP important for this trait were already present in set 1. BayesB and RKHS models performed equally well with predictions ranging from 0.331 to 0.582. Generally, these results suggest that imputation of missing SNP sites using the reference panel SNP has the potential to improve prediction accuracy but the increase depends on the trait architecture and the population under study.

**Table 1:** BayesB and RKHS prediction of stripe rust resistance for WWAM panel using SNP markers genotyped using 90K iSelect array, GBS and selected imputed markers with p-value ≤ 0.05 as informed by GWAS results

	BayesB model			RKHS model		
Traits	SNP set 1	SNP set 2	% gain	SNP set 1	SNP set 2	% gain
IT_mean	0.341 (0.019)	0.561 (0.023)	64.5	0.331 (0.020)	0.556 (0.023)	68.0
IT_BLUP	0.385 (0.021)	0.582 (0.021)	51.2	0.362 (0.020)	0.576 (0.021)	59.1
SV_Mean	0.348 (0.014)	0.532 (0.016)	52.9	0.343 (0.014)	0.534 (0.014)	55.7
SV_BLUP	0.350 (0.015)	0.542 (0.017)	54.9	0.350 (0.015)	0.540 (0.016)	54.3
SD	0.532 (0.013)	0.532 (0.017)	0.0	0.530 (0.015)	0.532 (0.017)	0.4

IT is infection type, SV is severity, SD is seedling resistance, SNP set 1 = 90K and GBS, SNP set 2 = 90K, GBS and imputed, % gain = 100*((SNP set 2 – SNP set 1)/ SNP set 1), values in parentheses are standard errors.

## Discussion

The power of IBD detection and the ability to accurately impute genotypes is influenced by the length of underlying IBD segments, the genotyping method utilized, and the method used for IBD analysis (Browning and Browning 2012). In experimental crosses or breeding populations based on a limited number of founders, significant length of IBD segments and their high population frequency allows for IBD detection using relatively modest marker density. In diverse populations, including distantly related lines separated by a large number of meiotic recombination events, the short length of IBD segments requires high marker density for their effective detection. In this study we have demonstrated that accurate genotype imputation using the re-sequenced reference panel of 62 wheat lines (Jordan *et al.* 2015) is feasible in a target population of wheat cultivars genotyped using the 90K SNP array or GBS. The imputation approach presented here can be applied to any wheat population previously genotyped using the 90K SNP iSelect array or GBS method. Our results showed that under a given imputation scenario the 90K SNP array dataset resulted in a higher fraction of imputed SNP than the GBS dataset. It is possible that the higher incidence of missing data in the GBS dataset and errors in mapping short reads to the complex wheat genome might have resulted in capturing a lower proportion of haplotype blocks in the WWAM panel compared to that captured by the 90K SNP iSelect array. Combining both GBS and 90K SNP resulted in a significant increase in the number of imputed genotypes, which increased the proportion of accurately predicted IBD segments. Improvements in the quality of the wheat genome reference sequence and increase in the accuracy of genotype calling from GBS data due to improvements in algorithms and increase in the depth of sequencing are expected to increase in the accuracy of IBD prediction and imputation. Further improvements can likely be achieved by using different imputations algorithms (Whalen *et al.* 2017, Shi *et al.* 2017) or, for cases when large reference panels are available, by applying reference selection algorithms that were shown to improve the accuracy of imputation (Shi *et al.* 2017).

The utility of genotype imputation using the reference panel of 62 wheat lines was further validated by performing association mapping and genomic prediction of stripe rust resistance in the winter wheat cultivars from the U.S. Great Plains. While the significant fraction (30%) of identified marker-trait associations overlapped with the previously mapped resistance loci (Fang *et al.* 2011; Basnet *et al.* 2014; Zegeye *et al.* 2014; Zurn *et al.* 2014; Daetwyler *et al.* 2014; Maccaferri *et al.* 2015), the population of cultivars from the Great Plains appears to carry previously unreported associations. Genotype imputation increased the number of markers available for GWAS of stripe rust resistance evidenced by a substantial number of significantly associated imputed SNP identified. The newly detected associations with the imputed SNP were in strong LD with the 90K array SNP and often showed more significant trait association than the SNP directly genotyped using the 90K SNP array. Stronger associations of imputed markers with a trait have been previously shown in human and *Arabidopsis* mapping projects (Abecasis *et al.* 2010; Cao *et al.* 2011). A more complete ascertainment of SNP in the regions linked with a trait increases the probability of detecting variants located on the same haplotype as the causal SNP, which improves the power and precision of association mapping studies.

Similarly, improvement in the prediction accuracy of infection type and severity of stripe rust was attributed to increased number of SNP imputed in the WWAM panel. Crossa *et al.* (2014) demonstrated the effect of increasing the number of SNP markers on the prediction accuracy of grain yield. However, several factors have been shown to affect the prediction accuracy of the models including genetic relatedness between the training and testing population, population size and genetic architecture of the trait (Bassi *et al.* 2016; Spindel *et al.* 2015; Wientjes *et al.* 2015). Our results suggest that selection of imputed markers based on their relative association with the trait reduced the noise of non-associated imputed markers leading to a great improvement in prediction accuracy of infection type and severity. Zhang *et al.* (2014) reported an improvement in whole genome prediction accuracy of complex traits by incorporating and giving markers weights that were associated with QTL based on previous GWAS results for the traits in dairy cattle and rice. However, they noted that the improvement in accuracy was dependent on the trait architecture. It was demonstrated in rice that for each trait there is a threshold on the number of markers required to achieve the highest prediction accuracy beyond which no gain is achieved by adding more markers (Spindel *et al.* 2015). Seedling resistance to stripe rust is known to be a monogenic trait (Aktar-Uz-Zaman *et al.* 2017). This could probably explain why there was no improvement in prediction accuracy by increasing the number of SNP in set 2. Infection type and severity are associated with adult plant resistance, which is polygenic in nature (Aktar-Uz-Zaman *et al.* 2017) hence, imputed SNP increased the chances of capturing most of the QTL linked to these traits. Indeed, BayesB model that accounts for additive genetic effect showed a slightly better prediction for the infection type and severity than seedling resistance as compared to RKHS model, which accounts for non-additive genetic effects (Pérez and de los Campos 2014).

Here we have demonstrated that by transferring genetic information from a densely genotyped reference panel of 62 wheat lines, we can significantly improve the efficiency of GWAS and genomic prediction. While the reference panel represents haplotypes not directly related to the U.S. winter wheat cultivars, it appears that both reference and target populations still share a significant fraction of chromosomal regions that are IBD. This observation is consistent with previous studies that demonstrated the low levels of genetic differentiation between global spring and winter wheat populations, and between populations of cultivars and landraces (Chao *et al.* 2010; Cavanagh *et al.* 2013). However, the lack of haplotypes representing the genetic diversity of U.S. winter wheat resulted in imputation of a smaller number of SNP in this study compared to that imputed in the population of spring wheat landraces (Jordan *et al.* 2015). As suggested in a study based on the analysis of 1000 human genomes (Howie *et al.* 2011), inclusion of additional reference haplotypes capturing the diversity of local populations is needed to significantly increase the number of accurately imputed SNP.

The reference panel of diverse wheat lines genotyped by exome capture re-sequencing represents a valuable resource for wheat genetics and breeding studies by providing a platform that allows transferring genomic variation data across multiple populations to increase the power and precision of trait mapping. Genomic prediction informed by results of GWAS greatly increased the prediction accuracy of traits. The value of this resource may be further improved by increasing the number of re-sequenced lines selected to capture the haplotypic variation of global wheat populations. In the future, this expanded reference panel would allow for the strategic selection of re-sequenced lines for imputation based on the relevance to the population of interest.
